# Biosynthesis and Functional Significance of Peripheral Node Addressin in Cancer-Associated TLO

**DOI:** 10.3389/fimmu.2016.00301

**Published:** 2016-08-09

**Authors:** Aliyah M. Weinstein, Walter J. Storkus

**Affiliations:** ^1^Department of Immunology, University of Pittsburgh School of Medicine, Pittsburgh, PA, USA; ^2^Department of Dermatology, University of Pittsburgh School of Medicine, Pittsburgh, PA, USA; ^3^University of Pittsburgh Cancer Institute, Pittsburgh, PA, USA

**Keywords:** high endothelial venule, L-selectin, peripheral node addressin, tertiary lymphoid organ, tumor

## Abstract

Peripheral node addressin (PNAd) marks high endothelial venules (HEV), which are crucial for the recruitment of lymphocytes into lymphoid organs in non-mucosal tissue sites. PNAd is a sulfated and fucosylated glycoprotein recognized by the prototypic monoclonal antibody, MECA-79. PNAd is the ligand for L-selectin, which is expressed on the surface of naive and central memory T cells, where it mediates leukocyte rolling on vascular endothelial surfaces. Although PNAd was first identified in the HEV of peripheral lymph nodes, recent work suggests a critical role for PNAd in the context of chronic inflammatory diseases, where it can be used as a marker for the formation of tertiary lymphoid organs (TLOs). TLO form in tissues impacted by sustained inflammation, such as the tumor microenvironment where they function as local sites of adaptive immune cell priming. This allows for specific B- and T-cell responses to be initiated or reactivated in inflamed tissues without dependency on secondary lymphoid organs. Recent studies of cancer in mice and humans have identified PNAd as a biomarker of improved disease prognosis. Blockade of PNAd or its ligand, L-selectin, can abrogate protective antitumor immunity in murine models. This review examines pathways regulating PNAd biosynthesis by the endothelial cells integral to HEV and the formation and maintenance of lymphoid structures throughout the body, particularly in the setting of cancer.

## Pathways Regulating PNAd Expression

### Signaling Through the Lymphotoxin Beta Receptor Is Required for HEV Differentiation

**Figure 1:**
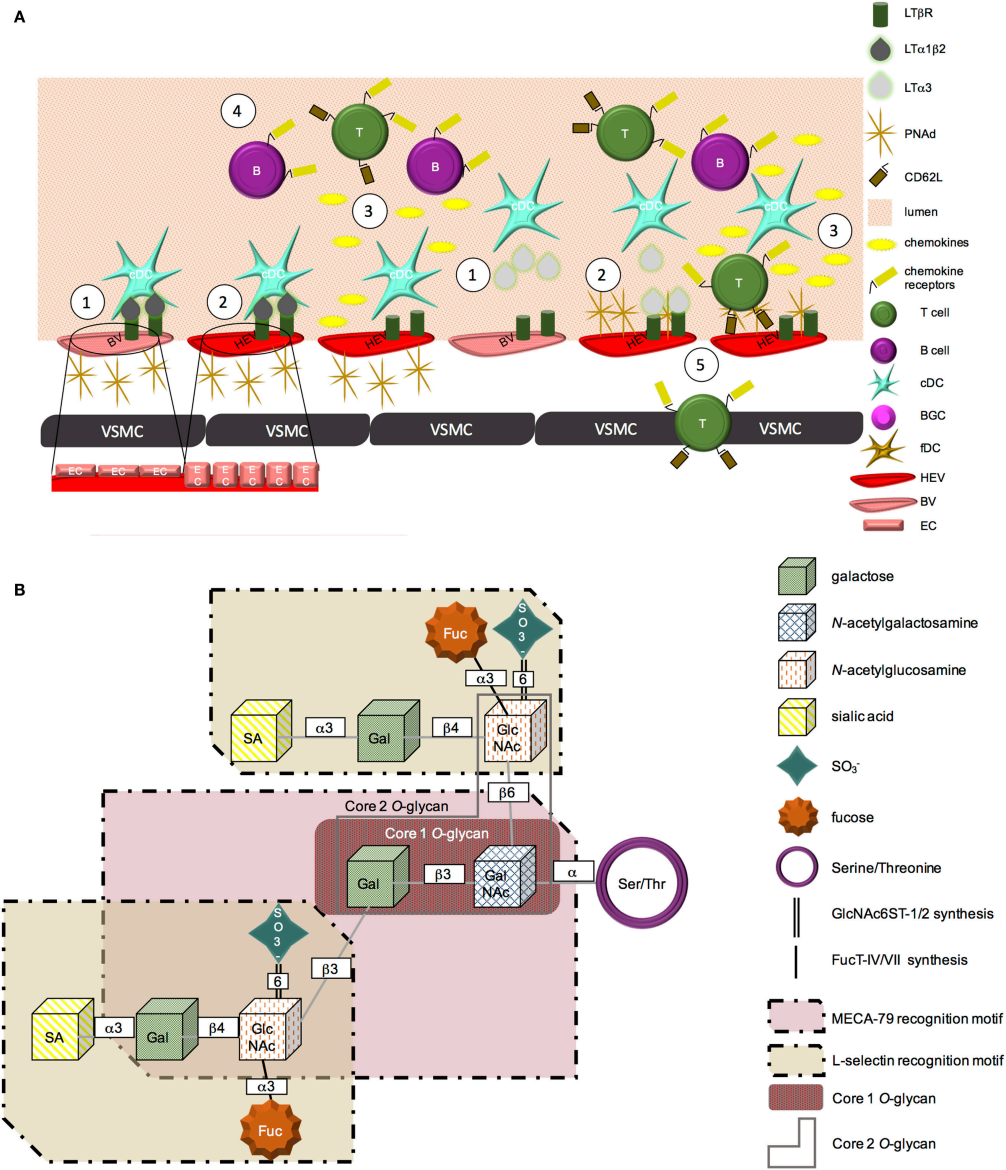
**PNAd biosynthesis**. **(A)** (1) LTβR is expressed on blood vessel endothelial cells. Membrane-bound LTα1β2 or secreted LTα3 secreted from cDC can signal through this receptor. (2) LTβR-mediated signaling promotes a physical change in vascular endothelial cells from a flat to cuboidal morphology. This signaling cascade also leads to the expression of PNAd on the surface of vascular endothelial cells, promoting HEV status. (3) LTβR signaling further induces HEV secreted chemokines, including CCL19, CCL21, and CXCL13. (4) Chemokines form gradients and “decorate” the blood vessel wall, initiating the recruitment of CCR7^+^ T cells or CXCR5^+^ B lymphocytes from the peripheral blood circulation into chronically inflamed tissues. (5) L-selectin on the surface of T cells is able to bind PNAd on the surface of HEV. These cells are then able to adhere to the vessel wall and extravasate into the tissue. **(B)** PNAd is synthesized from a Core 1 *O*-glycan. The extended Core 1 *O*-glycan serves as the MECA-79 recognition motif. The fucosylated Core 2 *O*-glycans are able to be recognized by L-selectin. Sulfation of the extended Core 1 and Core 2 *O*-glycans at the sixth position is mediated by GlcNAc6ST-1 and -2; α3 fucosylation is added by FucT-IV and -VII.

**Figure 2:**
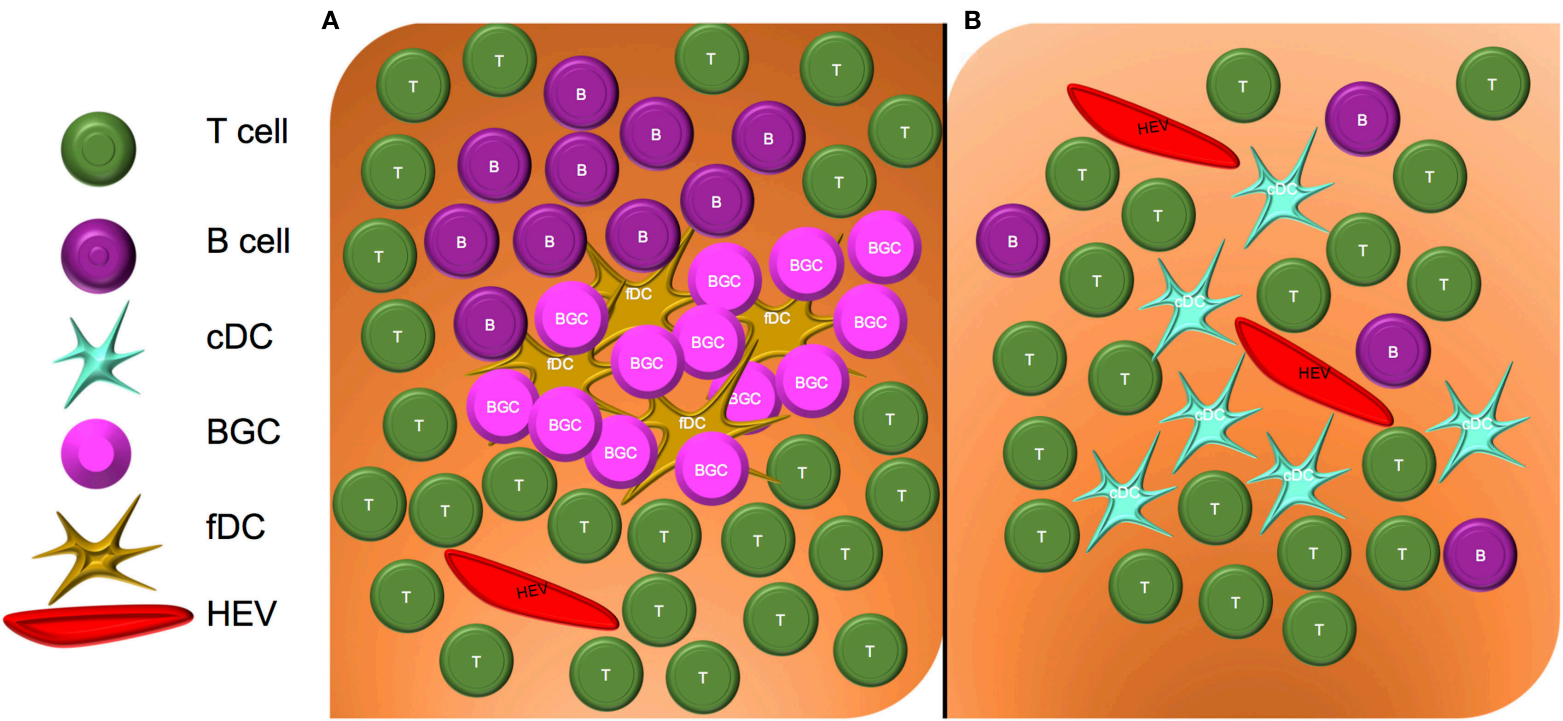
**Structure of classical and non-classical tertiary lymphoid organs**. **(A)** Classical TLO contain a nucleated core of fDC and germinal center B cells (BGC), surrounded by an HEV-containing T cell zone. **(B)** Non-classical TLO do not contain fDC or BGC, but may contain sparse B cell, T cell, and DC infiltrates surrounding HEV.

Lymphotoxin beta receptor (LTβR) signaling drives expression of adhesion molecules and chemokines involved in the recruitment of circulating lymphocytes into lymphoid organs, including CCL21, CXCL13, MAdCAM-1, and peripheral node addressin (PNAd) (1). Specifically, expression of LTβR on endothelial cells in peripheral lymph nodes is required for their development into high endothelial venules (HEV), with high endothelial cells (HEC) functioning as lymphoid tissue organizer (LTo) cells. Endothelial cell-specific deletion of LTβR leads to a reduction in: (i) MECA-79 staining, (ii) CCL19, CCL21, and GlyCAM-1 expression, and (iii) the ability to assume cuboidal morphology by endothelial cells in peripheral lymphoid organs [Figure 1A; (2)]. *In vivo* work using bone marrow chimeric mice deficient in LTα in their hematopoietic compartment also implicates a role for LTβR-mediated signaling in the maintenance of HEV, as these mice exhibit profoundly reduced lymph node cellularity (3).

LTα_1_β_2_ and LIGHT can bind and signal through the LTβR, while a related ligand, LTα_3_, can signal through TNFRI, TNFRII, and HVEM. All three ligands can be produced by CD11c^+^ DC (3). However, each ligand appears to have a distinct role in regulating PNAd expression. In secondary lymphoid organs (SLO), LIGHT appears to have little impact on PNAd expression (4). Using a transgenic model of lymphotoxin overexpression in the pancreas, it was observed that LTα and LTβ play distinct roles in the formation of tertiary lymphoid organs (TLOs). LTα_1_β_2_ controls luminal PNAd expression, while LTα_3_ controls abluminal PNAd expression (1). These differences in ligand function appear to relate to their impact on the level of GlcNAc6ST expression by endothelial cells. GlcNAc6ST-2 expression was reduced if only LTα, but not LTβ, was present, with HEV in LTβ^−/−^ animals (that retained GlcNAc6ST-2) expressing PNAd (1). LTα^−/−^ animals were deficient in GlcNAc6ST-2 expression on HEV, although they retained PNAd expression (5). Blockade of LTβR signaling also decreases transcription of GlcNAc6ST-2 in lymph nodes by 10-fold, with GlcNAc6ST-1, FucT-VII, and FucT-IV levels also coordinately reduced, thereby limiting posttranslational modification of PNAd and inhibiting its ability to be recognized by L-selectin (4).

Lymphocytes are also able to secrete lymphotoxin ligands (2, 4). Interestingly, the requirement for T or B cells themselves in HEV activation in SLO is equivocal. Reports suggest that neither cell type is required for HEV differentiation (4), although it has also been observed that *Rag*^−^/^−^ mice exhibit decreased expression of GlcNAc6ST-2 compared to WT mice (6).

### Posttranslational Modifications Are Required for L-Selectin Recognition of PNAd

Members of the PNAd family of addressins include GlyCAM-1, CD34, sgp200, podocalyxin, endomucin, and nepmucin: however, not all PNAd ligands appear to be required for lymphocyte trafficking (7, 8). For example, lymphocyte trafficking to peripheral lymph nodes remains unaltered in CD34^−/−^ (9) or GlyCAM1^−/−^ (10) mice, suggesting redundancy in the functional roles of PNAd family members. In order for PNAd to be recognized by MECA-79 as well as its receptor, L-selectin, a series of posttranslational modifications must first occur (Figure 1). Specifically, while PNAd undergoes sulfation and glycosylation (11), it is sulfation of the 6 sialyl Lewis X motif that renders these molecules recognizable by the MECA-79 antibody (12). Fucosylation of the Core 2 branched *O*-glycan serves as the recognition site of PNAd by L-selectin (Figure 1B) (13, 14).

#### Sulfation

GlcNAc6ST-1 and GlcNAc6ST-2 are members of the GalNAc6ST-6-*O*-sulfotransferase subfamily of glycosyl sulfotransferases that are critical to the transfer of sulfate groups to galactose or GlcNAc at the sixth position, with this sulfation of carbohydrate motifs on PNAd required for it to be presented at the cell surface and to be recognized by the MECA-79 antibody and by its natural ligand, L-selectin (15).

Though related, GlcNAc6ST-1 and GlcNAc6ST-2 have different roles in the sulfation of PNAd. Using mice deficient in either single sulfotransferase, it was shown that GlcNAc6ST-2 controls luminal expression of PNAd while GlcNAc6ST-1 controls expression of PNAd on the abluminal vascular surface (16, 17).

GlcNAc6ST-2 is expressed by mature, but not immature, HEV. Using a Cre-recombinase model, Kawashima and colleagues observed that expression of GlcNAc6ST-2 is activated in HEV cells recognized by the MECA-79 antibody (i.e., expressing PNAd), but not in cells reactive only with the MECA-367 antibody (recognizing MAdCAM-1) (18). This is consistent with observations that GlNAc6ST-1 and -2 have little impact on cellular expression of MAdCAM-1, a canonical marker of immature HEV in SLO within non-mucosal tissue sites (19).

#### Glycosylation

A family of α-(1,3)-fucosyltransferases control the fucosulation of E-, P-, and L-selectin ligands (20). In particular, FucT-VII and FucT-IV play distinct roles in the generation of L-selectin ligands on the surface of HEV. FucT-IV is required for the expression of L-selectin ligands on the surface of HEV, whereas the primary role of FucT-VII appears to be in its contribution to enhancing GlyCAM-1-mediated tethering of rolling lymphocytes. The specific role of FucT-VII temporally follows glycosylation and sulfation of the glycoprotein and is involved in capping the molecule to produce the preferred ligand recognized by L-selectin. Double knockout of both FucT-VII and FucT-IV in mice reduced lymphocyte recruitment to SLO by over 80% when compared to FucT-VII^−/−^ mice (21).

## Markers of High Endothelial Venules

Two sets of adhesion molecules dominantly modulate lymphocyte recruitment to SLO/TLO depending upon which site in the body the cells are trafficking to: recruitment to peripheral lymph nodes is dependent upon the L-selectin–PNAd interaction, while recruitment to mucosal sites requires the α_4_β_7_ integrin–MAdCAM-1 interaction (22). The same HEC that express PNAd or MAdCAM-1 also express CCL21, a CCR7 ligand. Supporting the importance of PNAd- and CCL21-expressing HEV for the recruitment of lymphocytes, the majority of lymphocytes in HEV-expressing tissues are spatially located within approximately 20 μm of HEV (23). CCL21 preferentially recruits CCR7^+^ CD4^+^ L-selectin^+^ (naive) T cells, which can interact with PNAd on the cells of the HEV. CCL21, like PNAd, is under the control of intrinsic LTβR-mediated signaling during HEV development (but not in mature lymphoid tissues) (1, 4).

## Immune Cell Recruitment by HEV

Peripheral node addressin binds L-selectin (aka CD62L or LECAM-1) expressed on the surface of lymphocytes. This interaction is required for the recruitment of lymphocytes into SLO (24). Posttranslational modifications of PNAd family members are critical for this interaction. For example, B cell recruitment to peripheral lymph nodes is dependent on sulfation of PNAd (19). The velocity of T and B cell rolling is also dependent upon sulfation of L-selectin ligands on lymph node endothelial cells, with adherence of lymphocytes to the vessel wall decreased in GlcNAc6ST-deficient animals (19). This may also be controlled by the presence of DC within SLO, as the velocity of lymphocyte rolling in CD11c-DTR mice was significantly increased, and the percentage of lymphocytes able to adhere to the vessel wall was decreased, in these mice after treatment with diphtheria toxin to delete DC. The HEV of DC-depleted mice regained expression of MAdCAM-1, and after reconstitution with adoptively transferred CD11c^+^ DC, these HEV recovered classical cuboidal morphology, suggesting that DC-produced factor(s) is/are required for the maturation of HEV (3).

The CCR7–CCL21 axis is also important for lymphocyte recruitment into lymphoid organs. Mice deficient in CCR7 have impaired migration of B and T cells, as well as DC, to SLO including lymph nodes and Peyer’s patches. This limits primary immune responses against infectious agents (25). Expression of CCL21 by HEC is controlled by a pathway unique to these cells versus HEC expressing alternate addressins. Specifically, heparan sulfate, a glycosaminoglycan primarily found on the surface of vascular endothelial cells, is required for CCL21 expression on HEV (26). Using an *Ext1*-flox/flox mouse crossed with a GlcNAc6ST-2-cre transgenic mouse to delete a glycosyltransferase necessary for the synthesis of heparan sulfate in PNAd-expressing cells, expression of CCL21 on the surface of HEV was abrogated (23).

## Tertiary Lymphoid Organs

Although the pioneering work identifying PNAd and the pathways controlling its expression were initially studied in the context of SLO, recent literature supports an important role for PNAd in TLO (aka ectopic lymphoid structures) that develop in peripheral tissue sites impacted by chronic inflammation. Overall, TLOs have varying degrees of similarity to SLO. Classical TLO closely resembles SLO in their cellular composition, with TLO containing a network of follicular dendritic cells (fDC) and germinal centers in which B cells reside, proliferate, and differentiate (Figure 2A). Non-classical TLO also contain some degree of B cell infiltration, but they do not exhibit an fDC “framework” (27), with only diffuse, sparse B cell distributions being observed (Figure 2B) (28–30).

The L-selectin–PNAd interaction controls lymphocyte recruitment to TLOs. In particular, PNAd upregulation in affected tissues is observed in the settings of allergic contact dermatitis, lymphoid hyperplasia, and a variety of types of skin lesions and cutaneous lymphomas, i.e., diseases characterized by robust lymphocytic infiltrates into peripheral tissues (31). The CCR7 signaling axis also plays a role in TLO formation. Most importantly, CCR7-mediated signals are required for the clustering of DC in peripheral tissues. Interactions between DC and T cells proximal to blood vessels appears required for the acquisition of PNAd + HEV in peripheral tissues (5).

Notably, LTβR-mediated signaling controls the formation of HEV in peripheral tissues (5, 32). Akin to the roles that lymphotoxin signaling plays in the control of PNAd expression in SLO, LTα3-dependent signaling has been reported to dominantly control PNAd expression on HEV within the tumor microenvironment (TME) in murine melanoma models (32), while in human breast cancer, LTβ (produced by DC-LAMP + DC) appears to play a comparable dominant role (33).

Tertiary lymphoid organs have been observed in a variety of chronic inflammatory diseases, including arthritis (34), gastritis and ulcerative colitis (13, 35), atherosclerosis (36), and cancer (37). As the development of TLO in chronic/autoimmune diseases has been well-reviewed (38, 39), we will now focus on the emerging field of TLO formation in solid tumors.

### TLO in Cancer

Cancer-associated TLO characteristically contain PNAd^+^ vessels and are commonly localized to the outer margin (versus the core) of the tumor lesion (40). With the exception of reports for TLO predicting a worse prognosis in patients with renal cell carcinoma (RCC) (41) and some cases of colorectal cancer (42), the vast weight of the literature has correlated the presence of TLOs in human solid tumors with better clinical prognoses (43). Both classical and non-classical TLO have been reported within the TME (Figure 2). Of these two forms of TLO, however, the presence of classical TLO in tumors may provide a superior index for improved prognosis when compared to the presence of only non-classical TLO in the TME (27). These results suggest that systematic analysis of PNAd expression and TLO status in tumor biopsies may be a useful in addition to current clinical criteria used to predict patient outcomes.

#### Lung Cancer

In non-small-cell lung cancer (NSCLC), PNAd^+^ vessels have been identified exclusively within TLO (44). In these tumors, the composition of cells within the TLO specifically correlates with patient prognosis. While T cells (all tumor-infiltrating L-selectin^+^ T cells, comprised of both naive and central memory CD4^+^ and CD8^+^ cells) are localized to TLO (44, 45), overall T cell infiltrate and density appears to play a minor role in patient outcome when evaluated independently of other prognostic markers. Instead, the density and proximity of mature DC to TLO within the tumor may be most important, and patients with high DC-LAMP^+^ mDC infiltrates exhibit markedly extended overall survival (45). These findings are further supported by gene array data indicating that *CXCR4*, a gene associated with DC migration toward CXCL12 gradients, is strongly correlated with increased overall survival in NSCLC patients (46). Unlike T cells, B cells do appear to play a significant protective role against lung cancer, and their presence can be used as a positive prognostic marker of overall survival. Interestingly, DC and B cell density in TLO can be used as a coordinate prognostic marker for patients with greatest overall survival. In NSCLC, B cells organize into germinal center-like structures containing CD21^+^ fDC. These B cells proliferate and differentiate *in situ*, leading to locoregional secretion of IgG and IgA antibodies reactive against tumor-associated antigens (47).

#### Skin Cancers

Tertiary lymphoid organs have been identified in both primary and metastatic melanoma, where they have been observed to contain PNAd^+^ vessels (48, 49). TLO in primary melanomas can be either classical or non-classical TLO. In metastatic melanoma, these structures are primarily composed of CD3^+^ T cells and mature (DC-LAMP^+^) DC proximal to PNAd^+^ HEV (50). Plasma B cells may also be present in such TLO, with these cells producing Th-dependent IgG and IgA antibodies specific for tumor-associated antigens (48, 50). In primary cutaneous melanoma, the presence of intratumoral HEV has been correlated with robust lymphocytic infiltration and tumor regression. Furthermore, if the HEC making up HEV have a cuboidal morphology, indicative of functional HEV, a positive correlation with CCR7, CCL19, and CCL21 expression within the tumor has also been observed (51).

The presence of TLO also portends better clinical outcome (recurrence free and overall survival) in the setting of Merkel cell carcinoma. These structures are also characterized by an increased CD8^+^/CD4^+^ T cell ratio at the tumor periphery and by a co-clustering of T and B cells within these anatomic sites (52).

#### Colon Cancer

Tertiary lymphoid organs in human colon cancer have been detected in both the colon crypt and at the invasive front of the tumor, as well as in the peritumoral region (53, 54). They contain immune cell types typically observed in SLO, including B cells, CD21^+^ fDC, T cells, and mature DC marked by DC-LAMP^+^, with CD31^+^ vascular endothelial cells and LYVE-1^+^ lymphatic vessels also noted (53, 54). T cells and mature DC represent positive prognostic markers in both primary (43) and metastatic (41) colorectal cancer. In such tumors, the B cells may not organize into germinal center-like structures (53, 54). These TLO appear to function as local sites for the priming and expansion of both B and T cells, based on the expression of the Ki-67 marker in *de facto* germinal centers in these diseased tissues (54).

#### Therapeutic Induction of TLO

Recent work from our group suggests that intratumoral TLO can be induced therapeutically *via* adoptive transfer of gene-modified DC, leading to reduced tumor progression. Following intratumoral injection of Type 1-polarized DC (DC engineered to overexpress Tbet, i.e., DC.Tbet) into established murine sarcomas or colon carcinomas, CD4^+^ and CD8^+^ T cell recruitment to the TME is observed within 2 days, with an upregulation of PNAd expression detected by 5 days after treatment. This suggests that PNAd-independent events control early T cell recruitment to the TME, and that T cell-dependent factors may consequently result in PNAd upregulation on tumor-associated VEC (28, 29). Once established, PNAd^+^ vessels become surrounded by dense infiltrates of both CD11c^+^ DC and CD3^+^ T cells, with these non-classical TLO principally localized near the tumor periphery for at least 2 weeks following initial therapeutic intervention (28, 29). The presence of DC in TLO is consistent with prior studies of SLO demonstrating that DC accumulation proximal to HEV is required for the subsequent optimal homing of lymphocytes into SLO (3).

## Future Perspectives

Although there appears to be some variability in the cellular composition across tumor types, TLO in the TME contain PNAd^+^ HEV typically surrounded by dense B cell and/or DC infiltrates. Importantly, the presence of intratumoral or peritumoral TLO has been almost universally linked with superior clinical prognosis in patients with solid forms of cancer. Though T cells are also present in intratumoral TLO, their presence has thus far proven equivocal as a prognostic biomarker. The spontaneous formation of TLO has been observed in a variety of human cancers, including those reviewed above as well as oral squamous cell carcinoma (27, 55), gastric cancer (40, 56), bladder cancer (57), breast cancer (30, 58, 59), and others (37, 60). Thus, it may ultimately be best to employ PNAd as well as B cell and DC infiltration in the TME as biomarkers to stratify patients based on TLO status, i.e., to differentiate individuals that may respond better to treatment intervention, including immunotherapies (based on superior locoregional immune competency). Furthermore, because TLO may be induced therapeutically (at least in murine models), it is also intriguing to speculate on the possibility that protective TLO may be conditionally sponsored in patients receiving chemo- or immunotherapies (61), and that such structures may be used to monitor/predict the patient’s outcome and prospective treatment management.

## Author Contributions

Both authors contributed to the design, writing, and editing of the submitted manuscript.

## Conflict of Interest Statement

The authors declare that the research was conducted in the absence of any commercial or financial relationships that could be construed as a potential conflict of interest.

## Abbreviations

BGC, germinal center B cell; BV, blood vessel; cDC, conventional dendritic cell; fDC, follicular dendritic cell; Gal, galactose; GalNAc, *N*-acetylgalactosamine; GlcNAc, *N*-acetylglucosamine; HEC, high endothelial cell; HEV, high endothelial venule; HVEM, herpes virus entry mediator; PNAd, peripheral node addressin; SA, sialic acid; SLO, secondary lymphoid organ; TLO, tertiary lymphoid structure; TME, tumor microenvironment; TNFR, tumor necrosis factor receptor; VSMC, vascular smooth muscle cell.
